# Comparison between stapled hemorrhoidopexy and harmonic scalpel hemorrhoidectomy in the management of third- and fourth-degree piles: a randomized clinical trial

**DOI:** 10.1007/s00104-023-02010-9

**Published:** 2023-12-29

**Authors:** Mohamed Ali Mohamed Nada, Philobater Bahgat Adly Awad, Andrew Morcos Azmy Kirollos, Mostafa Mohamed Abdelaziz, Karim Mohamed Saad Mohamed, Kerolos Bahgat Adly Awad, Basma Hussein Abdelaziz Hassan

**Affiliations:** General Surgery Department, Faculty of Medicine, Ain Shams University, 2022 Cairo, Egypt

**Keywords:** Hemorrhoids, Prolapse, Colorectal surgery, Bleeding, Surgical instruments

## Abstract

**Background:**

This study compared the results of stapled hemorrhoidopexy (SH) and harmonic scalpel hemorrhoidectomy (HSH) in the management of grade III and grade IV piles regarding the time of the procedure, postoperative pain, patient satisfaction, wound infection, bleeding, incontinence, and recurrence within 1 year.

**Patients and methods:**

This was a single-blind, prospective, randomized, controlled, single-center trial conducted from January to December 2022 that included 50 (68.75%) male and 20 (31.25%) female patients with third- and fourth-degree piles.

**Results:**

The patients were divided into two groups of 35 patients each. Group I underwent SH and group II underwent HSH. The mean age of group I was 42.94 years and of group II, 42.20 years. The mean time of the procedure was 24.42 min ± 2.367 for SH and 31.48 min ± 2.21 for HSH. Postoperative pain in group I was lower than in group II during the first 2 weeks, but there was persistent mild pain in most patients in group I at the 2‑week follow-up. In group II there was significant improvement in pain after 2 weeks, with higher patient satisfaction. Wound infection was detected in 3 (5%) patients in group I and no patients in group II (*p* = 0.077). Postoperative bleeding occurred in 4 (11.4%) patients in group I in the form of spotting after defecation only during the first postoperative month; no bleeding was detected in group II (*p* = 0.039). There were 3 (15%) cases of flatus incontinence but after taking a detailed history these were found to be cases of urgency to defecate rather than incontinence. There were 7 (20%) cases of recurrence at the 1‑year follow-up in group I and 1 (2.9%) case in group II (*p* = 0.024).

**Conclusion:**

Compared with SH, HSH was safer, easier, and associated with a lower incidence of recurrence after 1 year and with higher patient satisfaction.

## Introduction

The estimated worldwide prevalence of hemorrhoidal disease in adults is reported to be 11% [1]. In Egypt, hemorrhoid disease is a frequent reason for patients to seek medical attention. The incidence of hemorrhoids in patients undergoing colonoscopy was estimated to be 18% in Egypt [2]. There is currently a debate on how to classify hemorrhoidal disease, with many arguments in favor of Goligher’s classification. This is a descriptive classification of the characteristics of internal piles and distinguishes different groups based on the degree of prolapse. It provides a subjective representation of severity; however, it does not accurately portray the severity of symptoms [3]. There is currently no consensus on the use of the Goligher classification for hemorrhoids; however, in this study we aimed to grade the piles according to Goligher’s classification (see Table 1; [4]).

**Table 1:** Goligher classification for hemorrhoids

Grade	Degree of prolapse
I	No prolapse
II	Prolapse on defecation with spontaneous reduction
III	Prolapse on defecation requiring manual reduction
IV	Prolapse and irreducible

In the case of symptomatic grade III or grade IV hemorrhoids, surgical treatment is necessary. Additionally, surgery may be necessary in the case of failure of medical treatment or in the presence of associated conditions such as anal fissure or fistula. Different techniques are employed to treat hemorrhoidal disease, including conventional techniques such as Ferguson’s closed hemorrhoidectomy and Milligan–Morgan’s open hemorrhoidectomy. Additionally, a variety of devices and methods are employed to facilitate the procedure and reduce patient discomfort during the postoperative period. Stapled hemorrhoidopexy (SH) was first introduced by Longo in 1998 to be used in prolapsed hemorrhoidal disease. The goal of SH is not only to obliterate submucosal vessels, but also to return prolapsed rectal mucosa to its pre-prolapse state and to correct the anatomical relationship between the anorectal mucosa and the underlying muscle [5]. The harmonic scalpel was first introduced in 1992, and the goal of harmonic scalpel hemorrhoidectomy (HSH) is to use ultrasound as a source of energy, with minimal thermal damage to the surrounding tissue [6].

### Aim of this work

In this study we compared the results of SH and HSH in the management of grade III and grade IV hemorrhoids regarding the duration of the procedure and the postoperative outcomes in the form of postoperative pain, patient satisfaction, wound infection, postoperative bleeding, incontinence, and recurrence within 1 year.

### Patients and methods

The current study was a single-blind, prospective, randomized, controlled, single-center trial conducted from January 2022 to December 2022 in the colorectal surgical unit of Ain Shams University Hospitals. A total of 70 patients who presented with third-degree and fourth-degree piles were included—50 (68.75%) male and 20 (31.25%) female patients—and were followed up for1 year postoperatively.

### Ethical approval and consent to participate

To protect patient data and privacy, all methods were carried out in accordance with relevant guidelines and regulations and all experimental protocols were approved by Ain Shams University ethics committee with informed consent obtained from all the patients.

### Randomization and blinding

Randomization was performed on the day before surgery. Patients were randomized using a computer-generated randomization code and assigned either to experimental group I for SH or experimental group II for HSH. The two groups were balanced at a ratio of 1:1. The study was carried out under single-blind conditions.

### Inclusion criteria

Patients who were older than 18 years, with third-degree and fourth-degree piles, and with American Society of Anesthesiologists scores I and II were included in the study.

### Exclusion criteria

Patients who were younger than 18 years, with previous anal surgery or recurrent hemorrhoids were excluded. The following conditions were also considered to be exclusion criteria: patients with hemorrhoids accompanied by other anal conditions such as fissure, fistula, or anal condylomas; virgin female patients; inflammatory bowel disease such as Crohn’s disease; tuberculosis; and a history of fecal incontinence.

### Preoperative procedure

The history of the patients was taken including a full personal history and a list of complaints. A complete anorectal examination was performed and continence was assessed using the Wexner score.

The preoperative investigations included:

- Radiological examination: electrocardiogram (ECG) and echocardiography as well as stress ECG were performed upon request by the anesthesiologist when indicated.
- Laboratory tests: including routine complete blood count, liver profile, kidney profile, coagulation profile, blood sugar, and complete virology screen.

### Patient counseling and consent

One day before the surgery, the patient received a detailed explanation of the types of surgery and the expected postoperative complications. The operative details were explained to help the patient understand the outcome, the risks, and the benefits of the suggested procedure. An informed consent form was signed by the patient and any inquiries, concerns, or doubts were discussed with the patient and a first-degree relative (upon the patient’s request). On the day before surgery, all patients were instructed to have a soft diet and mineral laxative. The night before surgery, all patients had a rectal enema with ordinary tap water.

### Operative details

All procedures were performed by the same surgical team with the patient under spinal or general anesthesia in the lithotomy position. All patients received a single dose of 1 g of a third-generation cephalosporin intravenously at the induction of anesthesia.

#### Group I: stapler hemorrhoidectomy

**Fig. 1:**
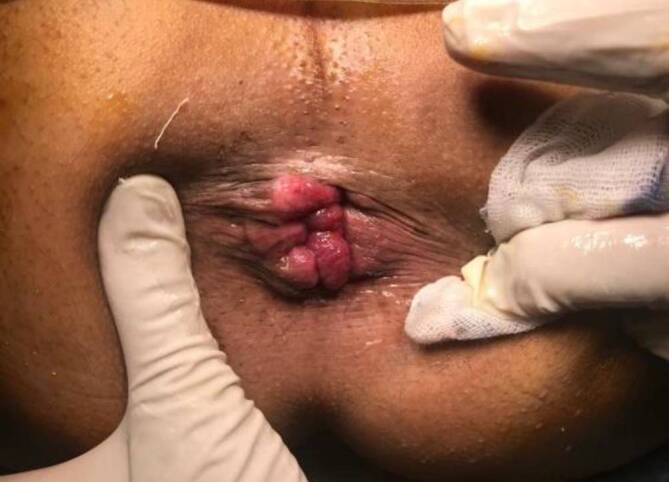
Fourth-degree piles in a patient in lithotomy position

**Fig. 2:**
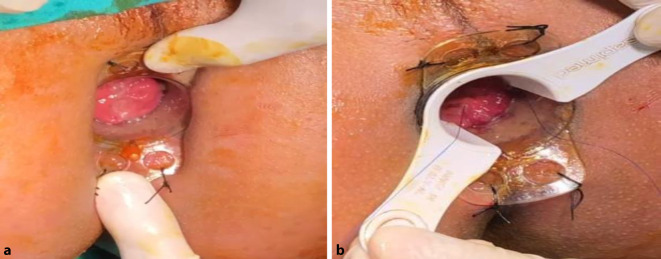
**a** The external device (transparent anoscope) of the PPH stapler is applied and fixed to the cutaneous margin with silk 0 sutures. **b** Double purse-string sutures inserted with submucosal bites of the lower rectum circumferentially using a 2/0 propylene suture

**Fig. 3:**
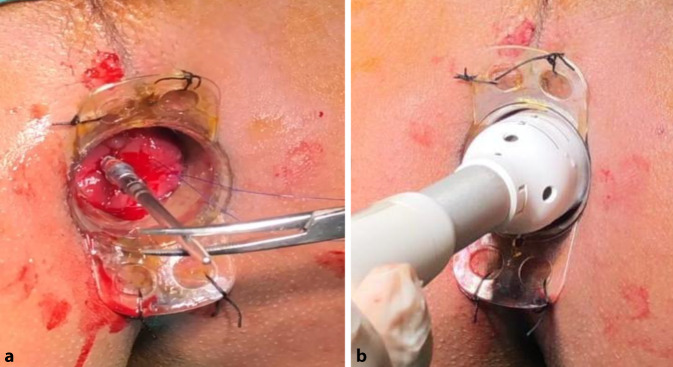
**a** The anvil (head) is inserted beyond the purse string. **b** The stapler is tied and kept closed for 60 s for hemostasis and then fired

**Fig. 4:**
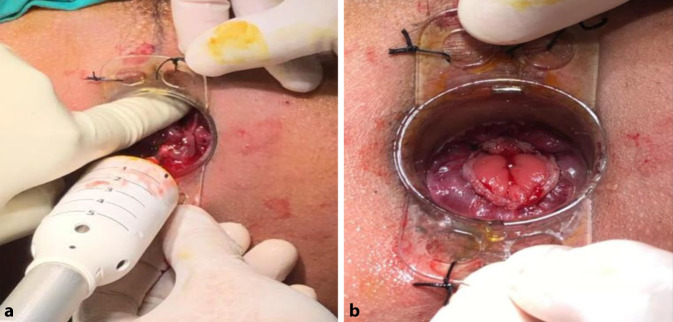
**a** In women, the posterior vaginal wall is routinely checked before firing the stapler to ensure non-entrapment. **b** Hemostasis is achieved along the stapler line

The external device (transparent anoscope) of a PPH stapler (PROXIMATE PPH Hemorrhoidal Circular Stapler Set; Ethicon Inc., Raritan, New Jersey, USA) was applied and fixed to the cutaneous margin with silk 0 sutures (Figs. 1 and 2a). Double purse-string sutures were inserted with submucosal bites of the lower rectum circumferentially using a 3/0 propylene suture (26-mm half-circle needle) at a level just a few millimeters distal to the apex of the anal cushions; the second purse-string was taken distal to the first one after applying a gentle pulling force on the first purse-string suture (Fig. 2b). The anvil (head) was inserted beyond the purse-string sutures, and the purse-string was then tied firmly over the stem of the anvil (the proximal one was tied first; Fig. 3a). The stapler was subsequently tied and kept closed for 60 s for hemostasis and was then fired (Fig. 3b). The next step was untying the stapler and gently withdrawing it. In women, the posterior vaginal wall was routinely checked before firing the stapler to ensure non-entrapment (Fig. 4a). Hemostasis along the staple line was achieved, and if required, a 3‑0 Vicryl suture was used in the case of bleeding (Fig. 4b).

#### Group II: harmonic scalpel hemorrhoidectomy

**Fig. 5:**
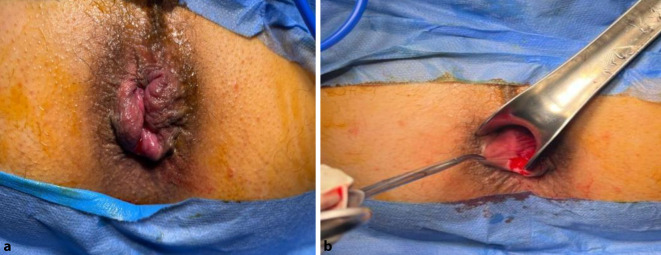
**a** Fourth-degree piles in patient in lithotomy position. **b** The mosquito forceps grasping a hemorrhoidal complex

**Fig. 6:**
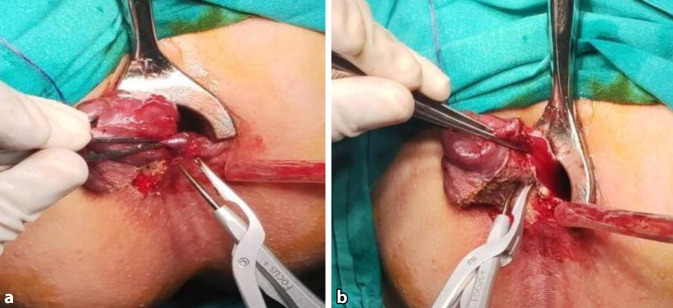
**a** The hemorrhoid is dissected from the surrounding tissue and underlying external sphincter by taking small bites between the device blades. **b** Proceeding more proximal underneath the hemorrhoid bundle, which is carefully separated from the internal anal sphincter

**Fig. 7:**
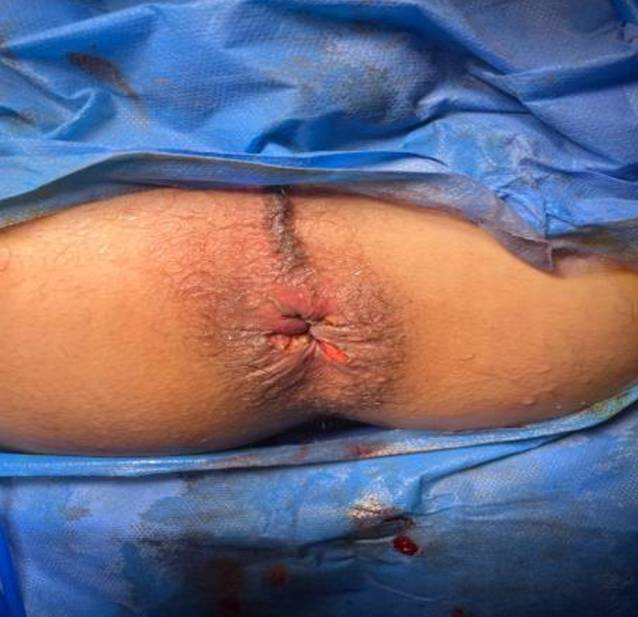
The same step as with the other hemorrhoids is carried out, leaving a skin bridge

A mosquito forceps or non-toothed forceps was used to grasp each hemorrhoidal complex (Fig. 5a, b). At the beginning, the external component of the hemorrhoid was dissected from the surrounding tissue and the underlying external sphincter by taking a small bite between the harmonic blades (Ethicon Endo-Surgery Inc., Cincinnati, OH, USA) and then proceeding more proximal underneath the hemorrhoid bundle, which was carefully separated from the internal anal sphincter using sequential coagulation with the harmonic scalpel blade on power mode 3 (Fig. 6a, b). The same steps were carried out with the other hemorrhoids, leaving a skin bridge between them (Fig. 7). Hemostasis was achieved using a cautery device and gel foam sponge. The wound was left open, and an external gauze was applied.

The postoperative work-up included a 1-year follow-up. Postoperative complications in the form of wound infection, postoperative pain, early incontinence, and recurrence were documented. Patients received oral antibiotics for 1 week postoperatively. Intake of liquid food was resumed in the evening after the operation and patients were advised to have a soft diet for 2 days and bulk laxatives for at least 2 weeks. Dressing of the wound was done on the second day postoperatively for all patients. All patients were trained on how to clean themselves and how to dress the wound. The patients were followed up at 1 week after the operation, 2 weeks from discharge, and then every 2 weeks until complete healing. Subsequently, patients were seen every 2 months to complete a 1-year follow-up. In this study recurrence is defined as patients reporting the reappearance of symptoms consistent with hemorrhoid symptoms or having to undergo surgery due to recurrent hemorrhoids. Patients were observed for recurrence during the follow-up period. None of our patients were lost to follow-up. It is noteworthy that none of the patients in either group was diagnosed with rectal or anal stenosis during the follow-up period.

### Statistical analysis

The data were collected, coded, revised, and entered into the Statistical Package for Social Science (IBM SPSS) version 26. Qualitative data are presented as number and percentages, and quantitative data with parametric distribution as mean, standard deviations, and ranges for the . The chi-square test was used to compare qualitative data between the two groups and Fisher’s exact test was used instead of the chi-square test when the expected count in any cell was less than 5. The comparisons between two independent groups with quantitative data and parametric distribution were made using an independent *t* test. The comparison between more than two groups with quantitative data and parametric distribution was made using a one-way ANOVA. The confidence interval was set to 95% and the margin of error accepted was set to 5%. The following statistical values were set: *p* > 0.05, nonsignificant (ns); *p* < 0.05, significant; *p* < 0.01: highly significant.

## Results

There were 70 patients in our study, with 35 patients in each group: 50 (68.7%) males and 20 (31.25%) females (*p* = 0.315). Group I underwent SH and group II underwent HSH. The mean age of group I was 42.94 years (SD: ± 11.8) and in group II it was 42.20 years (SD: ± 7.34; *p* = 0.315). Regarding the duration of the procedures, the mean time in the SH group was 24.42 min ± 2.367 while in the HSH group it was 31.48 min ± 2.21 (*p* = 0.186; Table 2). Table 2 shows the patients’ characteristics and the mean operative time between the two groups.

**Table 2:** Characteristics of the two patient groups and mean operative time

Characteristics	Group I (*n* *=* 35)	Group II (*n* *=* 35)	*p*
*Sex:*			0.016
Male (*n*) (%)	30 (85.7%)	20 (57.14%)	
Female (*n*) (%)	5 (14.2%)	15 (42.8%)	
*Mean age in years* *±* *SD*	42.94 ± 11.8	42.20 ± 7.34	0.315
*Comorbidities:*			0.54
Diabetes (*n*) (%)	7 (20%)	9 (25.7%)	
Hypertension (*n*) (%)	6 (17%)	9 (25.7%)	
Diabetes and hypertension (*n*) (%)	4 (11.4%)	5 (14.2%)	
No comorbidities (*n*) (%)	18 (51.4%)	12 (34.2%)	
*Mean operative time in mins* *±* *SD*	24.428 ± 2.37	31.485 ± 2.2145	0.186

Postoperative pain was assessed in two stages: during the first 2 weeks and after the first 2 weeks on the basis of the Visual Analogue Scale (VAS), with scores ranging from 0 to 10, with 0 being the lowest and 10 the maximum. During the first 2 weeks: In group I, 18 (45%) patients scored their pain as mild from 1 to 3, 20 (50%) patients scored their pain as moderate from 4 to 6, and two (5%) patients scored their pain as severe from 7 to 9. In group II, 14 (35%) patients scored their pain as mild from 1 to 3, 22 (55%) patients scored their pain as moderate from 4 to 6, and four (10%) patients scored their pain as severe from 7 to 9 (*p* = 0.275; Table 3).

**Table 3:** Postoperative pain according to VAS scores (first 2 weeks)

			Groups		Total
			HSH	SH	
Postoperative pain VAS (first 2 weeks)	Mild pain	Count	10	20	30
		% postoperative pain VAS (first 2 weeks)	33.3%	66.7%	100.0%
		% within groups	28.6%	57.1%	42.9%
	Moderate pain	Count	20	15	35
		% postoperative pain VAS (first 2 weeks)	57.1%	42.9%	100.0%
		% within groups	57.1%	42.9%	50.0%
	Severe pain	Count	5	0	5
		% postoperative pain VAS (first 2 weeks)	100.0%	0.0%	100.0%
		% within groups	14.3%	0.0%	7.1%
Total		Count	35	35	70
		% postoperative pain VAS (first 2 weeks)	50.0%	50.0%	100.0%
		% within groups	100.0%	100.0%	100.0%

*SH* stapled hemorrhoidopexy, *HSH* harmonic scalpel hemorrhoidectomy, *VAS* Visual Analog Scale

Although, postoperative pain in patients who underwent SH was lower during the first 2 weeks, there was persistent mild postoperative pain in most of these patients. However, in those who underwent HSH, there was significant improvement in pain after 2 weeks and, therefore, patient satisfaction was significantly higher in the HSH group (Table 4). Table 4 shows the postoperative pain scores between the two groups.

**Table 4:** Postoperative pain according to VAS scores (after 2 weeks)

			Groups		Total
			HSH	SH	
Postoperative pain VAS (after 2 weeks)	No pain	Count	14	15	29
		% postoperative pain VAS (after 2 weeks)	48.3%	51.7%	100.0%
		% within groups	40.0%	42.9%	41.4%
	Mild pain	Count	17	11	28
		% postoperative pain VAS (after 2 weeks)	60.7%	39.3%	100.0%
		% within groups	48.6%	31.4%	40.0%
	Moderate pain	Count	4	9	13
		% postoperative pain VAS (after 2 weeks)	30.8%	69.2%	100.0%
		% within groups	11.4%	25.7%	18.6%
Total		Count	35	35	70
		% postoperative pain VAS (after 2 weeks)	50.0%	50.0%	100.0%
		% within groups	100.0%	100.0%	100.0%

*SH* stapled hemorrhoidopexy, *HSH* harmonic scalpel hemorrhoidectomy

Wound infection was detected in three (5%) patients in group I and no patient in group II (*p* = 0.077). Postoperative bleeding occurred in four patients in group I in the form of spotting after defecation, but it was only experienced during the first postoperative month, while no patient in group II was affected by this (*p* = 0.039). There were no cases of incontinence in group II. In group I, there were three (15%) cases of flatus incontinence only, according to the Wexner score of 3/20 (*p* = 0.026), but after taking a detailed history these were found to be cases of urgency to defecate rather than incontinence. There were seven cases (20%) of recurrence at the 1‑year follow-up in group I and one case (2.9%) in group II (*p* = 0.024). These results are presented in Tables 5**,** 6 and 7.

**Table 5:** Postoperative bleeding

			Groups		Total
			HSH	SH	
Postoperative bleeding	No	Count	35	31	66
		% postoperative bleeding	53.0%	47.0%	100.0%
		% within groups	100.0%	88.6%	94.3%
	Yes	Count	0	4	4
		% postoperative bleeding	0.0%	100.0%	100.0%
		% within groups	0.0%	11.4%	5.7%
Total		Count	35	35	70
		% postoperative bleeding	50.0%	50.0%	100.0%
		% within groups	100.0%	100.0%	100.0%

*SH *stapled hemorrhoidopexy, *HSH *harmonic scalpel hemorrhoidectomy

**Table 6:** Postoperative incontinence

			Groups		Total
			HSH	SH	
Incontinence Wexner score	Perfect	Count	35	32	67
		% incontinence Wexner score	52.2%	47.8%	100.0%
		% within groups	100.0%	91.4%	95.7%
	Sometimes	Count	0	3	3
		% incontinence Wexner score	0.0%	100.0%	100.0%
		% within groups	0.0%	8.6%	4.3%
Total		Count	35	35	70
		% incontinence Wexner score	50.0%	50.0%	100.0%
		% within groups	100.0%	100.0%	100.0%

*SH *stapled hemorrhoidopexy, *HSH *harmonic scalpel hemorrhoidectomy

**Table 7:** Rate of recurrence

			Groups		Total
			HSH	SH	
Recurrence	No	Count	34	28	62
		% recurrence	54.8%	45.2%	100.0%
		% within groups	97.1%	80.0%	88.6%
	Yes	Count	1	7	8
		% recurrence	12.5%	87.5%	100.0%
		% within groups	2.9%	20.0%	11.4%
Total		Count	35	35	70
		% recurrence	50.0%	50.0%	100.0%
		% within groups	100.0%	100.0%	100.0%

*SH *stapled hemorrhoidopexy, *HSH *harmonic scalpel hemorrhoidectomy

**Fig. 8:**
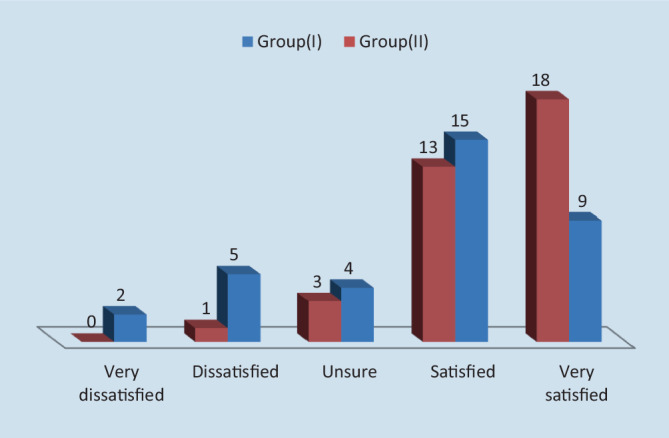
Postoperative patient satisfaction

Postoperative patient satisfaction was assessed after 2 weeks using a 5-point Likert scale: 1=very dissatisfied, 2=dissatisfied, 3=unsure, 4=satisfied, and 5=very satisfied. In group I there were two (5.7%) patients who were very dissatisfied, whereas in group II there were no cases of very dissatisfied patients (*p* = 0.15). In group I, five (14.3%) patients were dissatisfied and in group II there was one (2.9%) dissatisfied patient (*p* = 0.198). In group I, four (11.4%) patients were unsure, while in group II there were three (8.6%) patients who were unsure (*p* = 0.69). In group I, 15 (42.9%) patients were satisfied and in group II this number was 13 (37.1%; *p* = 0.8). Finally, in group I, nine (25.7%) patients were very satisfied, while in group I there were 18 (51.4%) very satisfied patients (*p* = 0.049; Table 8 and Fig. 8).

**Table 8:** Postoperative patient satisfaction assessed on a Likert scale

	Very dissatisfied *n* (%)	Dissatisfied *n* (%)	Unsure *n* (%)	Satisfied *n* (%)	Very satisfied *n* (%)
Group I (*n* = 35)	2 (5.7%)	5 (14.3%)	4 (11.4%)	15 (42.9%)	9 (25.7%)
Group II (*n* = 35)	0 (0%)	1 (2.9%)	3 (8.6%)	13 (37.1%)	18 (51.4%)
*p*	0.15	0.198	0.69	0.8	0.049
Total *n*, (%)	2 (2.9%)	6 (8.6%)	7 (10%)	28 (40%)	27 (38.6%)

## Discussion

Patient satisfaction is a major factor in the surgeon’s decision-making process and is the primary reason why piles surgery is not a preferred procedure for the majority of surgeons. However, it has been observed that the success of any procedure is not the only factor that influences postoperative patient satisfaction: Patient satisfaction can be affected by many factors such as patient perception. Recent studies have indicated that adequate postoperative pain management can lead to increased patient satisfaction [7]. However, there is still a need for more information on patient satisfaction specifically in regard to the various types of piles surgery. This study attempted to compare the effectiveness of SH versus HSH.

The comparison between the two methods revealed no significant difference in the mean duration of the procedure: 24.42 min ± 2.367 in the SH group and 31.48 min ± 2.21 in the HSH group (*p* = 0.186). The fact that SH can be performed by an experienced coloproctology surgeon may be why the duration of SH was shorter. However, it does not negate the fact that SH requires a skilled hand due to the technical nature of the procedure. In our study HSH was found to be slower, which may be attributed to the fact that it works on each pile individually, thus varying in time depending on the number of piles. Pain was assessed at two stages by asking patients about the pain in the initial 2 weeks and at 3 months post-surgery. Stapled hemorrhoidopexy is well known as one of the least painful methods in pile surgery [8]. Chung et al. [6] compared the two techniques in their study of 88 patients, and found that the SH group reported significantly lower VAS postoperative pain, reduced hospitalization, quicker return to normal activities, and increased patient satisfaction. Contrary to other studies, such as the study by Bilgin et al. [9], mean total pain scores did not achieve statistical significance. In our study, there was a significant difference in pain during the initial 2 weeks. However, the most notable finding was the dramatic improvement in pain after 2 weeks in the HSH group, with persistent mild pain reported during the 3 months postoperatively in the SH group.

Postoperative complications were significantly increased in the SH group, with bleeding being one of the most significant short-term complications. Previous comparative studies between the two techniques were inconclusive, with Chung et al. [6] reporting a lower incidence of postoperative bleeding for SH, which is in contrast to the majority of studies in the literature. Bilgin et al. [9] reported less postoperative bleeding with the HSH technique, while other studies, such as that by Armstrong et al. [10], found no short-term postoperative bleeding. In the literature, the postoperative bleeding rate for SH ranges from 4% to 26% [11–13], while for HSH it ranges from 0% to 4% [9, 10, 14]. In our study, postoperative bleeding was present in four patients in the SH group in the form of spotting with defection; in two patients it persisted for 3 months.

In this study, there were no reported cases of wound infection in the HSH group while three cases of wound infection were found in the SH group: two of infected perianal hematoma during the first month and one case of intersphincteric collection 2 months after surgery. In the literature, infection is generally agreed to be a rare complication in HSH [9, 15], while in SH various types of wound infection have been described. The first case of death due to wound infection was reported by Bohnner et al. [16], and since then, numerous studies [17–22] have reported other septic complications due to SH. It is not possible to draw a definitive conclusion on the prevalence of wound infections in SH; however, it is generally accepted that these complications can be life-threatening.

There were no cases of incontinence in the HSH group because during the procedure the metal plate of the harmonic scalpel enables good dissection and identification of the internal sphincter, thereby avoiding its injury, which is a great advantage of this technique. In a recent meta-analysis [23] that compared nine types of piles surgery, HSH was found to have the lowest incontinence rate. However, in the SH group in our study there were three cases of flatus incontinence (Wexner score of 1–3/20), which is a significant difference between the two techniques. There is high variability regarding the rate of incontinence in SH in the literature. This variability, in our opinion, is related to the complexity of the technique; Bilgin et al. [9] reported a 2% rate of flatus incontinence with SH. Michalik et al. [24] conducted a study to assess long-term outcomes after SH. In their study, 21% patients and 11% patients presented with flatus incontinence and fecal incontinence, respectively, which is considered a very high rate and may reflect a type of bias.

In the literature there is consensus in favor of HSH when comparing recurrence rates between the two procedures. In a meta-analysis conducted by Aibuedefe et al. [8], HSH was found to have one of the lowest recurrence rates when compared to 12 other techniques. Additionally, Talha et al. [25] claimed no recurrence rate in the HSH group. On the other hand, a number of studies discussed recurrence rates of SH, with some authors reporting recurrence rates as high as 60%, for example, Zacharakis et al. [26], and others reporting more reasonable rates, such as Jayaraman et al. [27], who found 23 recurrences in a group of 269 patients undergoing SH. At the end of a 2-year follow-up, Bilgin et al. [9] reported recurrence in seven patients undergoing SH (13.7%) with only one case of recurrence in their HSH group (2.1%). In our study, we found two cases of recurrence among the HSH patients and seven cases of recurrence among 35 patients (8.6%) in the SH group.

Patient satisfaction was considered to be one of the most significant differences between the two techniques in our study. As this point has not been extensively discussed previously, it is important to note that patient satisfaction is dependent on the surgeon’s preference for a technique and his experience using it. In our study, patient satisfaction was observed to be higher in the HSH group. According to Chen et al. [28], the number of patients who were poorly satisfied or dissatisfied was 121 of 321 patients undergoing SH. In the study by Chung et al. [29], comparing the outcome of patients undergoing hemorrhoidectomy with harmonic scalpel, with bipolar scissors, or with the Milligan–Morgan technique, the authors concluded that HSH had the highest satisfaction score of the three methods.

## Conclusion

It is important to note that there is no one-size-fits-all approach to the management of grade III and grade IV piles. Each technique has its own advantages and disadvantages, and each patient is presented with a unique situation: They must choose between SH, with less postoperative pain and a greater likelihood of recurrence and increased complications, or HSH, with lower recurrence rates and increased safety but with higher postoperative pain in the first 2 weeks after surgery. In our study, HSH was determined to be safer, easier, and associated with a lower incidence of recurrence at the 1‑year follow-up compared with the SH technique. In addition, there is better patient satisfaction, which is one of the greatest challenges in the management of hemorrhoids. This work recommends HSH as a cornerstone procedure along with the various other classic operations due to its ease of use and feasibility.

## Data Availability

The datasets used and/or analyzed during the current study are available from the corresponding author on reasonable request.

## Declarations

*Ethical approval and consent to participate: *To protect patient data and privacy, all methods were carried out in accordance with relevant guidelines and regulations and all experimental protocols were approved by the Ain Shams University ethics committee with informed consent obtained from all the patients. *Trial no:* PACTR202310528525409, *Date of Approval:* 31/10/2023.
